# Note on Croton-Chloral-Hydrate

**Published:** 1874-10

**Authors:** I. Ott

**Affiliations:** Corresponding Member of Atlanta Academy of Medicine, Easton, Pa.


### NOTE ON CROTON-CHLORAL-HYDRATE.

By I. OTT, M.D.,
*Corresponding Member of Atlanta Academy of Medicine, Easton, Pa.*

Some of this substance coming into our hands, we thought it highly important that the observations detailed by Liebreich* should be again examined by experimental evidence.

It is a white crystaline body, with chloral-like odor; disagreeble taste; soluble with difficulty in cold water; pretty soluble in hot water, and extraordinarily soluble in alcohol. It was discovered a few years ago by Kramer and Pinner. The following is the observation of Leibreich: In its primary stage it produces sleep, strong anæsthesia of the parts of the head supplied by the trigeminal nerve; in its secondary stage it destroys all reflex excitability, leaving the pulse and respiration intact, and finally causes death by paralysis of the medulla oblongata. Electrical irritation of the central end of the pneumogastric in the poisoned animal shows no contraction of the diaphragm. Excitation of phrenic causes it to contract. Immense doses produce cardiac paralysis. Artificial respiration will save poisoned animals. In man it produces deep sleep—anæsthesia of the parts supplied by the trigeminus, whilst the sensibility of the body persists; the pulse and respiration remaining unaltered. In neuralgia of the face, and other painful affections of the same part, it has been found useful by Leibreich, J. Wickham Legg, H. C. Wood and Benson Baker. Dr. Wood saw ten grains of the drug produce decided relief in a case of tic of central origin. I experimented

---

*Wrigger's und Bleusemauri's Jahresbeucht, 1871. Wood, H. C., Toxicology.

on frogs and rabbits; making only one experiment on a cat and one on myself. Our croton-chloral hydrate was obtained from Merck, and was given suspended in syrup.

Experiment 1.—Frog received, at 9:45 A.M., .0025 gramme of croton-chloral-hydrate subcutaneously. At first he hopped away, then remained quiet. 10 A.M., cornea exhibits considerable anæsthesia, hopped when touched, recovered completely during the afternoon.

Experiment 2.—Frog, at 1:7 P.M., received .005 gramme of poison, subcutaneously. At first hops about. 1:10 P.M., sat still, respiratory movements now and then. 1:12 P.M., sensibility of cornea abolished, but when its feet were pinched, hopped away, dozing; respirations twelve per minute. 1:22 P.M., anterior extremities have lost their mobility, posterior extremities weak, hops with difficulty. 1:40 P.M., cornea insensible, general sensibility persisting, struggles when pinched. 2 P.M., sits quietly dozing. 5 P.M., slight mobility and sensibility. Died during the night.

Experiment 3.—Frog, at 10:30 A.M., received .01 gramme of croton-chloral-hydrate, subcutaneously; right iliac artery previously tied; hops away. 10:32 A.M., cornea becoming insensible; now and then retching movements; sits quietly. 10:42 A.M., frog sleeping; cornea nearly insensible; anterior extremities weak; posterior extremities pretty strong. 10:48 A.M., cornea insensible; when animal is pinched he crawls away. 11 A.M., reflex action persisting in all the extremities. 11:20 A.M., complete loss of mobility and sensibility; respiratory movements at intervals of a minute; chest opened; heart-beat forty-six per minute; motor nerves of tied and poisoned extremities do not seem to have lost much motricity; no reflex action or excitation of the central ends of sciatic. 2:40 P.M., reflex action manifest; cornea insensible; .04 gramme subcutaneously injected. 2:45 P.M., no respiration; when needle is thrust into spine, slight tremulous muscular movements.

Experiment 4.—Frog, at 11:34 A.M., received 1.6 grammes subcutaneously. At first leaps away, then sits still; fore extremities become weak; abdominal posture; motility lessened; sensibility of cornea lost; general sensibility persisting. 11:40 A.M., when extremities are pinched he hops away; no respiration visible. 11:45 A.M., abdominal posture; cornea insensible. 12 M., no sign of life; chest opened; heart making a few weak movements; motor nerves irritable at two and a half centimetres Dubois coil and one Seclanche cell; thrusting needle into spinal cord causes a few weak movements; heart in diastole, filled with dark blood; abdominal viscera normal; muscles at point of injection discolored.

Experiment 5.—Rabbit, at 11 A.M., received .32 gramme of croton-chloral hydrate subcutaneously. Moves about; sits still. 11:12 A.M., rabbit seems dozing; ears congested; makes a few rotary movements; seems rather unsteady. 11:17 A.M., slight tremor of muscles about the jaws; respirations seem diminished. 12 M., rabbit sits quietly; sensibility of cornea much diminished; rabbit seems sleepy; general sensibility and motility persisting; cardiac and respiratory movements do not seem to be much interfered with; recovers during the afternoon.

Experiment 6.—Rabbit received, at 4:50 P.M., .64 gramme of croton-chloral-hydrate subcutaneously. Runs about; frequent respiration; sitting quietly; tremors about muscles of the jaw. 5 P.M., cornea nearly insensible; general sensibility and motility good; sleeping. 5:5 P.M., when pinched moves away; ears congested. 5:7 P.M., pupil contracted; respirations less frequent. 5:12 P.M., cornea insensible. 5:17 P.M., sways to one side, as though losing equilibrium during sleep; sensibility and motility of extremeties good. 5:25 P.M., same state. 5:45, dozing; recovered during the night.

Experiment 8.—A little kitten received .32 gramme subcutaneously at 9:30 A.M. 9:32 A.M., falls over on one side; tongue protruded. 9:35 A.M., cornea insensible; some sensibility and motility in the trunk; respiration twenty per minute; all reflex action abolished; pupil at first contracted, now dilated. 9:40 A.M., cat dead; chest opened; heart beating feebly and slowly; motor nerves excitable; a needle thrust into spine causes no muscular movements.

Experiment 9.—I took .32 gramme of croton-chloral-hydrate at 1:22 P.M. I seemed to lapse into forgetfulness at 1:35 P.M. 1:40 P.M., again took .32 gramme. 1:45 P.M., sensibility of face considerably lessened; it feels stiff; corneal sensibility also less. 2 P.M., I took 1:60 grammes. 2:35 P.M., sensibility of face and cornea very much diminished. 2:40 P.M., feel sleepy. 3:30 P.M., feel very sleepy, dozing a short time; can pass finger over cornea without pain; general sensibility and motility good; respiratory and cardiac apparatus does not seem disturbed; walk about without any difficulty. 6:30 P.M., sensibility of face and cornea nearly normal.

In the above experiments the march of symptoms seems to be as follows: Trigeminal anæsthesia, sleep, diminution of motility and sensibility of the body, loss of reflex excitability, death by paralysis of respiratory centers, and, with large doses, cardiac arrest. It is seen that considerable anæsthesia of the trigeminus can be produced without much impairment of sensory and motor functions of the rest of the body. It does not (in large medicinal doses) prove a dangerous cardiac depressant like chloral. The muscular relaxation of chloral is also absent. It has occurred to us during these experiments that it might be useful to oculists in operations on the eye, as extraction of foreign bodies, etc. Reasoning from a physiological stand point, this body bids fair to supersede chloral where there is a cardiac contraindication for the latter, and to palliate the horrors of facial neuralgia.

